# Tissue Taurine Depletion Alters Metabolic Response to Exercise and Reduces Running Capacity in Mice

**DOI:** 10.1155/2014/964680

**Published:** 2014-11-12

**Authors:** Takashi Ito, Natsumi Yoshikawa, Stephen W. Schaffer, Junichi Azuma

**Affiliations:** ^1^Department of Clinical Pharmacogenomics, School of Pharmacy, Hyogo University of Health Sciences, Kobe, Hyogo 650-8530, Japan; ^2^Department of Pharmacology, University of South Alabama College of Medicine, Mobile, AL 36688, USA

## Abstract

Taurine is a sulfur-containing amino acid found in very high concentration in skeletal muscle. Taurine deficient mice engineered by knocking out the taurine transporter gene exhibit skeletal muscle wasting, structural defects, and exercise intolerance. In the present study, we investigated the mechanism underlying the development of metabolic abnormalities and exercise intolerance in muscle of the TauTKO phenotype. Running speed and endurance time of TauTKO mice were lower than those of control mice. Blood lactate level was elevated by >3-fold during treadmill running in TauTKO mice but remained largely unaltered by exercise in WT mice. Blood glucose was cleared faster during treadmill running in TauTKO mice than WT mice. AMP-activated kinase (AMPK) *β*-2 subunit was reduced in TauTKO muscle concomitant with a reduction in *α*1 and *α*2 subunits of AMPK. The level of PPAR*α* and its targets, Gpx3, Cpt2, and Echs1, were also decreased in TauTKO muscle. Collectively, taurine depletion impairs metabolic adaptation to exercise in skeletal muscle, a phenomenon associated with a downregulation of AMPK and diminished NADH utilization by the mitochondrial respiratory chain. These findings suggest a crucial role of taurine in regulating energy metabolism in skeletal muscle of exercising TauTKO mice, changes that contribute to impaired exercise endurance.

## 1. Introduction

Taurine, a sulfur-containing amino acid found in very high concentration in mammalian tissues, is particularly high in skeletal muscle. Recent evidence supports a role of taurine in exercise endurance. Mice lacking the taurine transporter (TauT) gene and containing severely reduced muscle taurine content exhibit exercise intolerance in both treadmill and forced swimming tests [1, 2]. Moreover, taurine supplementation prolongs the time to exhaustion during treadmill running, an effect accompanied by the release of intramuscular taurine into the blood [3, 4]. Furthermore, taurine treatment diminishes skeletal muscle cramping in patients with myotonia and liver cirrhosis [5, 6]. However, the mechanism underlying the effect of taurine on exercise endurance has not been clarified.

Taurine is considered an essential nutrient in species, such as cat and fox, which exhibit little capacity to synthesize taurine but require large amounts of taurine to maintain normal levels of conjugated bile acids. By comparison, although rodents contain the enzymes for taurine biosynthesis in the liver, maintenance of the large intracellular taurine pool in muscle depends upon uptake of taurine from extracellular space via a taurine transporter (TauT). Disruption of the TauT decreases the taurine content of skeletal muscle by more than 98% [1, 2]. Furthermore, TauT is induced during myotube differentiation in C2C12 myoblasts [7], suggesting a critical role of TauT in maintaining homeostasis of mature muscle.

It has been revealed that taurine possesses a variety of biological actions that potentially alter contractile function, such as modulation of ion movement, osmoregulation and regulation of oxidative stress [8, 9]. In skeletal muscle, taurine regulates excitation-contraction coupling by modulating Cl-conductance [5, 10]. Furthermore, we have previously demonstrated that TauTKO mice display muscle wasting and histological muscular defects, such as myofibril derangement and appearance of autophagic bodies [1, 11]. These structural defects are consistent with the impairment of exercise capacity.

Muscle intolerance has often been associated with impairment in energy metabolism [12]. Therefore, it is significant that taurine deficient hearts from rats fed the taurine transport inhibitor, *β*-alanine, show elevated rates of glucose uptake and glycolysis resulting in enhanced rates of lactate production [13]. Crossover analysis has revealed that three events regulate glycolysis in taurine deficiency, stimulation of phosphofructokinase, limited flux through glyceraldehyde-3-phosphate dehydrogenase, and acceleration of lactate formation from pyruvate, consistent with a rise in NADH/NAD^+^ ratio.

Recently, Kirino et al. and Schaffer et al. reported that taurine serves as a substrate for the enzyme catalyzing the formation of 5-taurinomethyluridine tRNA^Leu(UUR)^ in mitochondria. Because the conjugation product enhances the interaction between the codon and anticodon of tRNA^Leu(UUR)^, mitochondrial taurine content regulates the biosynthesis of mitochondria encoded proteins and hence the activity of respiratory chain complexes [14–16]. According to Jong et al., taurine deficient neonatal cardiomyocytes in culture exhibit diminished rates of oxygen consumption, an effect consistent with stimulation in lactate production [17]. By contrast, Warskulat et al. have reported an increase in the respiratory quotient (*V*_CO_2__/*V*_O_2__) of TauTKO mice during treadmill exercise, despite an elevation in muscle lactate content at sedentary and diminished muscular performance [2]. Although not discussed, the findings of Warskulat et al. suggest that efficiency of the TauTKO mouse is diminished, as the exercising TauTKO mouse exhibits enhanced energy metabolism despite impaired capacity to exercise. We reasoned that, at higher exercise loads, the demand for energy production could exceed the capacity of muscle to produce ATP, leading to further impairment of muscle performance. To test this idea, we analyzed the metabolic response to treadmill running in TauTKO and wild-type mice. We also examined changes in the gene expression profile of TauTKO skeletal muscle, changes that reflect an adaptation to the metabolic stress imposed on the TauTKO mouse from alterations in energy inefficiency.

## 2. Methods

### 2.1. Animal Care

All experimental procedures were approved by the Institutional Animal Care and Use Committee of Hyogo University of Health Sciences. TauTKO and littermate mice (C57BL/6) were housed in SPF environment and fed a standard chow (MF, Oriental Yeast, Japan), had access to water ad libitum, and were maintained on a 12 h light/dark cycle. Female WT and TauTKO mice were studied. Since we have previously reported that several phenotypes, including muscle histology, swimming tolerance, cardiac function, and longevity, were similar between WT and hetero mice, we used them as control for TauTKO mice [1, 18].

### 2.2. Treadmill Experiments

**Figure 1:**
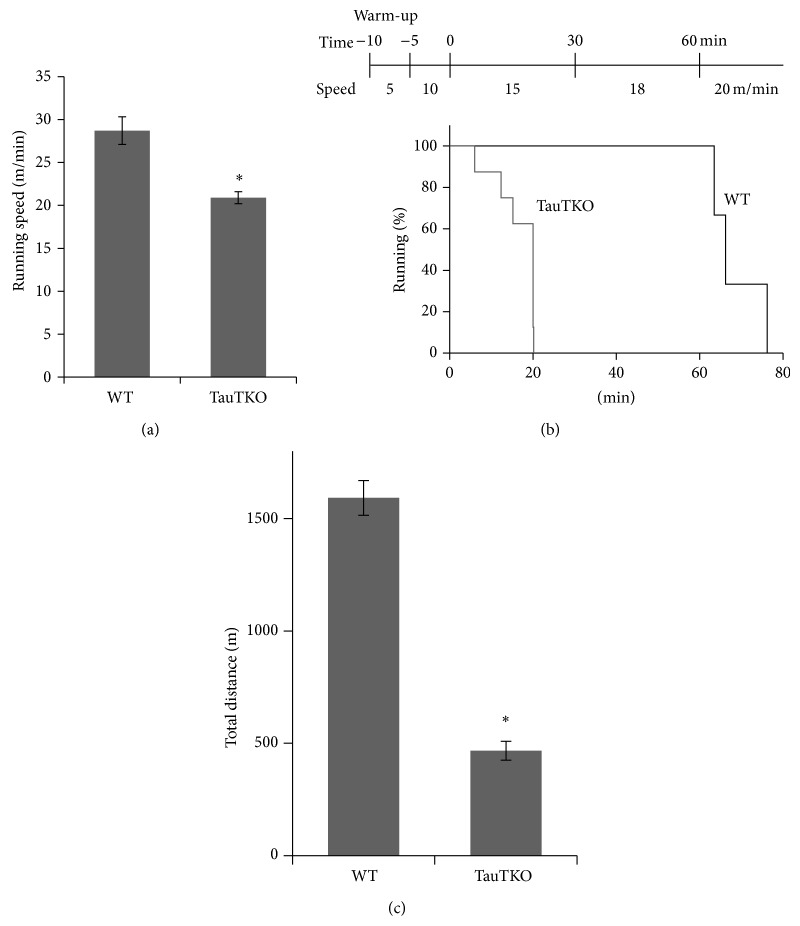
*Running speed and running duration in TauTKO and WT mice.* (a) Mean running speed of TauTKO (*n* = 8) and WT mice (*n* = 6). (b) Running endurance of WT and TauTKO mice (TauTKO: *n* = 8, WT: *n* = 3). This graph shows the survival plot indicating the percent of WT and TauTKO mice running at the indicated time during treadmill running test (15–20 m/min, 0% gradient). Lines mean percent of WT mice (black) and TauTKO mice (gray). Total run distance was calculated in (c).

For the speed test, 4- to 6-month-old mice were initially subjected to a warm-up protocol consisting of 5 min running on the treadmill at 5 m/min, 0 degrees, followed by 5 min at 10 m/min, 0 degrees. After the warm-up period, the running speed on the treadmill was increased by 1 m/min every 30 sec (Natsume Seisakusho, Japan) until the mice stopped running. An electrical stimulation (30 V) was applied on the shock grid. During the exercise tolerance test, the warm-up period was followed by forcing mice to run at 0 degrees until exhaustion, at which point they ceased running. During preliminary experiments, both male and female TauTKO mice exhibited lower exercise capacity. However, since male KO mice failed to run on the treadmill adequately for any reason, only female mice were utilized to determine exercise capacity and metabolic response. The running speed during most running protocols (see Figure 1(b)) was initially set at 15 m/min but after 20 min was increased to 18 m/min followed by an additional 30 min at 20 m/min. To examine the effect of exercise on energy metabolism, 4~9-month-old mice ran at 15 m/min for 20 min to evaluate the effect of exercise prior to exhaustion (TauTKO: *n* = 15, WT: *n* = 9). Prior to and following completion of the exercise protocol, a drop of blood was obtained from tail vein and blood glucose and lactate were measured using Precision Xceed (Abbot Japan, Japan) and Lactate Pro (Arkray, Japan). After running, mice were immediately killed by guillotine and tissue was rapidly isolated and frozen in liquid nitrogen and stored at −80°C. Sedentary mice with the same month age were also killed and tissue was isolated (TauTKO: *n* = 11, WT: *n* = 13).

### 2.3. Metabolite Measurements

For the measurement of lactate, pyruvate, and ATP, muscle was homogenized in 10% perchloric acid and centrifuged and supernatant was neutralized with 1 M NaHCO_3_. Lactate and pyruvate were measured by minor modifications of previously described enzymatic fluorometric methods [19, 20]. Briefly, the lactate assay buffer was added to supernatant in a 96-well plate followed by incubation at 37°C for 1 h. Fluorescence was detected by Spectraq Max M2 (Molecular Devices) at 460 nm emission (with 340 nm excitation). The assay mixture consisted of 100 mM hydrazine, 100 mM glycine (pH 9.1), 0.5 mM NAD^+^, and 14.4 U/mL lactate dehydrogenase (Toyobo). For lactate determination, pyruvate assay buffer was added to supernatant in a 96-well plate that had been incubated at room temperature for 30 min. Fluorescence was measured at settings of 530 nm excitation and 590 nm emission. The assay mixture consisted of 100 mM potassium phosphate (pH 5.9), 1.0 mM EDTA, 10 mM MgCl_2_, 10 *μ*M FAD, 10 mM thiamine pyrophosphate, 50 *μ*M AmplexUltrared (Invitrogen), 0.5 U/mL pyruvate oxidase, and 5 U/mL horseradish peroxidase (Toyobo). ATP content was assayed using a commercial kit bioluminescent method according to manufacturer's protocol (Roche). Luminescence was detected by Spectra Max L luminometer (Molecular Devices).

For the glycogen measurement, muscle and liver isolated from mice were lysed in 3 M potassium hydroxide at 100°C and neutralized by 1 M sodium acetate. After precipitation, glycogen was degraded by Amidase (Sigma) in 0.5 M acetate buffer (pH 4.0) and glucose units were determined by the glucose oxidase method (Wako Pure Chemicals, Japan).

### 2.4. Quantitative Reverse Transcript PCR

Total RNA was isolated from tibial anterior skeletal muscle of TauTKO and wild-type mice using Sepazol (Nacalai tesque, Japan) according to the manufacturer's protocol. Total RNA (1 *μ*g) was subjected to reverse transcription with Rever Tra Ace (Toyobo, Japan). Quantitative RT-PCR analyses were performed by using Applied Biosystems Step One Plus (Applied Biosystems) with THUNDERBIRD SYBR qPCR Mix (Toyobo, Japan). The primers used are shown in Table 1. *β*-Actin (*actb*) was used as an internal control.

### 2.5. Western Blot

Tibial anterior muscle was homogenized in RIPA buffer (10 mM Tris-HCl, pH 8.0, 150 mM NaCl, 1 mM EDTA, 1% nonidet-40, 1% deoxycholate, protease inhibitor cocktail (Nacalai tesque), phosphatase inhibitor cocktail (Nacalai tesque)) using a Potter homogenizer and then sonicated on ice for 15 s at 10% amplitude with 1 s on/off intervals using a microtip sonicator (Branson Sonifier). After centrifugation, the protein concentration of the supernatant was determined by the BCA assay. SDS sample buffer (x2: 125 mM Tris-HCl, pH 6.8, 4% SDS, 10% Mercaptoethanol, 20% Glycerol, 0.002% Bromophenol Blue) was added to the lysate. Western blot was performed by the semidry method as described previously [21]. Anti-AMPK sampler kit, anti-phospho-acetyl CoA carboxylase Ser79 (Millipore), and anti-GAPDH antibody (Chemicon, CA) were used.

### 2.6. Statistics

Each value was expressed as the mean ± standard error (SE). Statistical analysis was performed using Statcel 2nd edition (OMS Publishing Inc). Student's *t*-test or Turkey-Kramer test was used to determine statistical significance between groups. Differences were considered statistically significant when the calculated *P* value was less than 0.05.

## 3. Results

### 3.1. Exercise Capacity Is Impaired in TauTKO Mice during Treadmill Running Test

To evaluate the effect of taurine deficiency on exercise capacity, running speed and running duration of TauTKO and control mice were determined. During the treadmill running test, TauTKO mice displayed a 27% reduction in running speed (Figure 1(a)). Moreover, exercise endurance duration was drastically decreased in TauTKO mice (Figures 1(b) and 1(c)).

### 3.2. Metabolism during Treadmill Exercise in TauTKO Muscles

**Figure 2:**
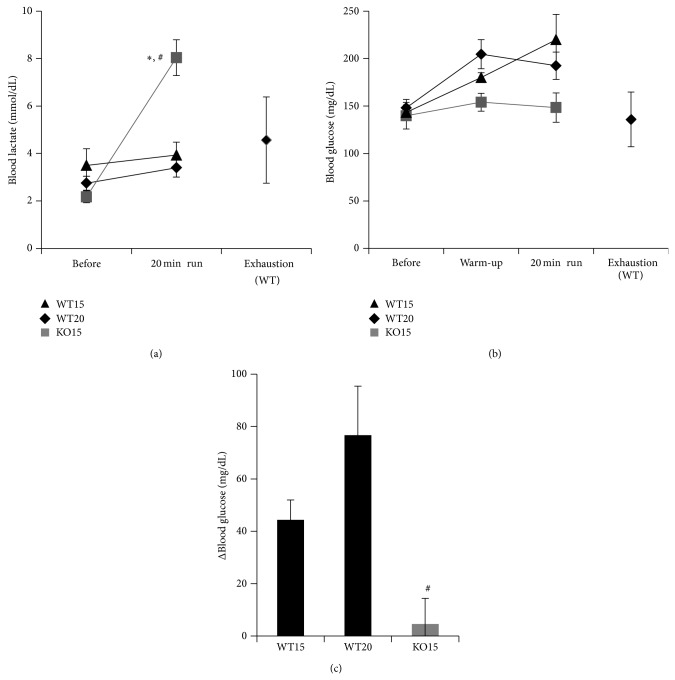
*Alterations in blood lactate and glucose concentration during exercise in TauTKO and WT mice.* (a, b) Blood lactate (a) and glucose concentration (b) were measured at the indicated time points when mice were running on the treadmill at 10 m/min for 10 min (warm-up) at 15 m/min or 20 m/min (WT mice only) for 20 min (20 min run). Black triangles: WT mice who ran at 15 m/min (WT 15); black diamonds: WT mice who ran at 20 m/min (WT20); gray squares: TauTKO mice who ran at 15 m/min. (c) The changes of blood glucose before and after 20 min running were calculated. Data are mean ± se. *n* = 3–7. ^*^ *P* < 0.05 versus WT, ^#^ *P* < 0.05 versus before running.

To assess the effect of exercise on glucose metabolism of TauTKO mice, the levels of blood lactate and glucose were measured before and after treadmill running (Figures 2(a)–2(c)). Although lactate levels remained unchanged after the 20 min running at 15 m/min in WT mice, they were significantly elevated in TauTKO mice that either completed the 20 min running protocol or ran until exhaustion. Enhancing the running speed (20 m/min) had little effect on blood lactate levels of the WT mouse (Figure 2(a)). The mild degree of exercise during the warm-up period caused a modest increase in blood glucose levels in WT mice, probably because of stress-induced elevations in gluconeogenesis and glycogenolysis [22]. By contrast, the warm-up period had no effect on blood glucose levels in the TauTKO mouse (Figures 2(b) and 2(c)).

The rise in blood lactate after a 20 min running procedure at a speed of 15 m/min is only partially attributed to the difference in glucose handling between WT and TauTKO mice, as the differential in blood lactate and blood glucose levels between the two mouse groups was >4 mmol/dL and 0.2 mmol/dL, respectively (Figures 2(a) and 2(c)). Because these parameters were identical prior to exercise, one explanation for the enhanced accumulation of lactate in the blood of the TauTKO mouse (relative to the WT mouse) is the preferential production of lactate from glycolysis. Alternatively, enhanced mobilization of glycogen could deliver more glucose to the glycolytic pathway of the TauTKO mouse, thereby leading to an elevated rate of lactate production. To distinguish between these two possibilities, the levels of lactate, pyruvate, and glycogen were measured in gastrocnemius muscle of WT and TauTKO mice during rest and following exercise.  Table 2 shows that the lactate/pyruvate ratios, which are proportional to the NADH/NAD^+^ ratios, of gastrocnemius muscle of WT mice prior to and following running were similar (lactate/pyruvate ratios were ~78). By contrast, the lactate/pyruvate ratio of the gastrocnemius muscle of TauTKO mice increased from 56 to 113 during the transition from rest to running. Thus, the elevation in blood lactate in the exercising TauTKO mouse is related in part to an increase in the NADH/NAD^+^ ratio of skeletal muscle that diverts pyruvate into lactate at the expense of pyruvate oxidation by the mitochondria. The rise in the NADH/NAD^+^ ratio also indicates inefficient generation of ATP, also reflected by the decline in gastrocnemius ATP levels of the exercising TauTKO mice (Table 2).

Taurine deficiency had no significant effect on either hepatic or gastrocnemius glycogen levels before or following exercise. Hence, exercise-mediated mobilization of skeletal muscle glycogen is similar in WT and TauTKO mice. Moreover, the mobilization of glycogen in the liver, although sufficient to elevate plasma glucose levels, was similar in the two groups of mice (Table 2). Thus, while exercise-mediated glycogenolysis contributes to lactate production by the gastrocnemius, the contribution is identical in the two groups of animals, suggesting that the differential in blood lactate content between the WT and TauTKO mice is caused by preferential lactate generation secondary to the rise in the NADH/NAD^+^ ratio.

### 3.3. AMPK Expression in TauTKO Muscle

**Figure 3:**
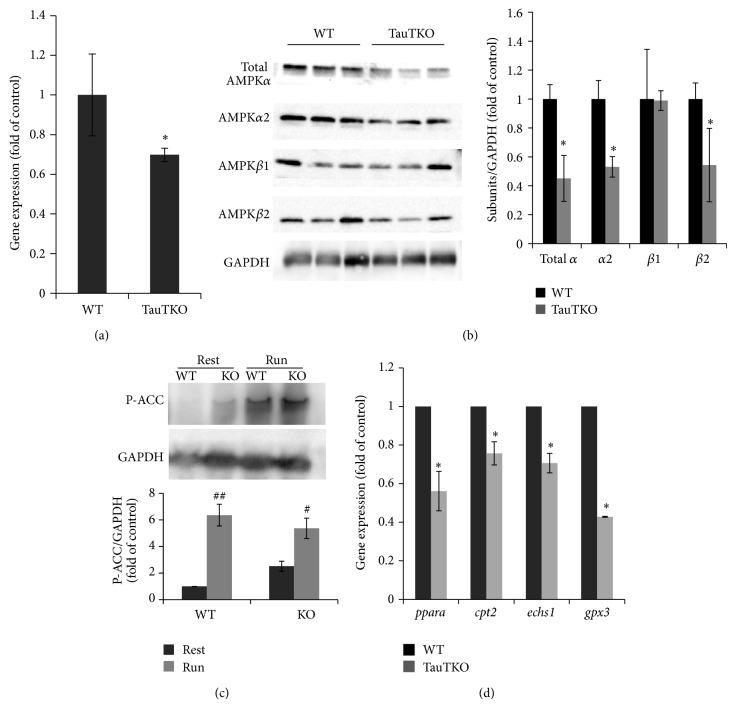
*Expression of AMPK subunits and genes of PPAR*α* and its targets in tibial anterior muscle of WT and TauTKO mice.* (a) Gene expression of AMPK *β*2 was measured by qRT-PCR method. *n* = 4. ^*^ *P* < 0.05 versus WT. (b) AMPK subunits (*α*1, *α*2, *β*1, and *β*2) and GAPDH in TA muscle of WT and TauTKO mice were detected by Western blot. *n* = 3. ^*^ *P* < 0.05 versus WT. (c) Phospho-ACC (Ser79) and GAPDH in skeletal muscle before and 20 min after treadmill running were detected. Data are mean ± se. *n* = 3–6. ^#^ *P* < 0.05, ^##^ *P* < 0.01 versus rest group. (d) The change in genes of PPAR*α* (ppara), carnitine palmitoyl transferase 2 (cpt2), short-chain enoyl CoA hydratase (echs1), and glutathione peroxidase c (gpx3) was analyzed by quantitative RT-PCR. Data are mean ± SE. *n* = 4–7. ^*^ *P* < 0.05 versus WT.

The observation that taurine deficiency stimulates lactate production and glycogenolysis while downregulating the enzymes of fatty acid metabolism suggests that the competition between fatty acid and glucose metabolism is shifted in favor of glucose metabolism in the TauTKO mouse. These changes occur without an alteration in ATP levels, although there is a modest reduction in ATP content of skeletal muscle of exercising TauTKO mice. Because AMP-activated kinase (AMPK) is both a sensor and regulator of energy metabolism, the expression of AMPK in TauTKO skeletal muscle was examined. Based on real-time PCR experiments, the expression of the *β*-2 subunit of AMPK (AMPK*β*2) was downregulated in skeletal muscle of TauTKO mice (Figure 3(a)). In agreement with the PCR results, Figure 3(b) shows that the protein content of AMPK*β*2 was also reduced in TauTKO muscle. Accompanying the downregulation of AMPK*β*2 was a reduction in AMPK*α*1 and AMPK*α*2 content. However, we failed to detect the difference of beta 1 subunit level between WT and TauTKO. One of the proteins regulated by AMPK is acetyl CoA carboxylase (ACC) [23], an enzyme indirectly regulating the transport of long chain fatty acids into the mitochondria for *β*-oxidation. Interestingly, the phosphorylation state of ACC of TauTKO muscle was enhanced under basal, resting conditions, a change resulting in increased activity of the enzyme. The degree of phosphorylation of ACC was also enhanced during running, with the rise in phosphorylation reaching levels that were ~6-fold control levels in both WT and TauTKO mice, a change also noted in the AMPK*β*2 knockout model [24].

One of the key regulators of fatty acid metabolism is PPAR*α*, which is controlled by the *β*2 subunit of AMPK [23]. Because several genes involved in fatty acid metabolism are downregulated in TauTKO skeletal muscle containing reduced AMPK*β*2 levels, the expression of peroxisome proliferator-active receptor-*α* (PPAR*α*) were examined. As seen in Figure 3(d), the expression of PPAR*α* was lower in skeletal muscle of TauTKO muscle than in WT muscle. Moreover, the expression of several enzymes involved in lipid metabolism (Cpt2, Echs1, and Gpx3), which are putative PPARalpha targets, was also downregulated in TauTKO skeletal muscle. These included a key regulator transporter (carnitine palmitoyltransferase 2), which is required for the transfer of long chain fatty acids into the mitochondria. An enzyme involved in *β*-oxidation of fatty acids (enoyl CoA dehydratase) was also downregulated by taurine deficiency. Together, these findings indicate that the metabolism of fatty acids is suppressed in TauTKO skeletal muscle.

## 4. Discussion

The present study shows that TauTKO mice exhibit impaired exercise capacity, as both maximal running speed and duration are diminished relative to that of WT mice (Figure 1). Taurine depletion is also associated with muscle cramping and exercise-induced muscle injury [5, 25]. However, the mechanisms underlying these pathological changes in muscle performance remain to be elucidated.

It has been reported that taurine is released from muscle during exercise, an effect associated with an increase in muscle fiber osmolality related to the accumulation of metabolic by-products, such as lactate [26]. In the present study, we demonstrated that after 20 min exercise plasma lactate was significantly increased in TauTKO mice but not in WT mice. Exercise also mediated a net increase in the lactate/pyruvate ratio of gastrocnemius muscle of the TauTKO mouse but not that of the WT mouse. Because the lactate/pyruvate ratio is a measure of the cytosolic NADH/NAD^+^ ratio, the results suggest that a defect in NADH utilization by the mitochondria results in a rise in the NADH/NAD^+^ ratio of the cytosol, which leads to the conversion of pyruvate to lactate rather than the utilization of pyruvate by the citric acid cycle. A similar effect is seen in the taurine deficient, beating heart [13].

Glycolysis is a major source of ATP during skeletal muscle contraction. In TauTKO muscle, ATP production is affected by a shift in metabolism away from fatty acids in favor of glycolysis. Figure 3(d) shows that several genes involved in the oxidation of long chain fatty acids are downregulated in TauTKO muscle. These same genes (Gpx3, Echs1, and Cpt2) are regulated by AMP kinase (AMPK) and PPAR*α* [27–29], which are also downregulated in TauTKO muscle. Also suppressing fatty acid metabolism is impaired flux through the respiratory chain, which increases the NADH/NAD^+^ ratio, resulting in the inhibition of key citric acid cycle and *β*-oxidation dehydrogenases involved in fatty acid metabolism [30]. It is noteworthy that respiratory function is also depressed in AMPK*α*2 and AMPK*β*1*β*2 knockout mice [22, 31, 32]. Thus, the shift away from fatty acid *β*-oxidation to glucose metabolism in TauTKO muscle may be caused in part by the downregulation of AMPK*α*2 and AMPK*β*2 and the corresponding decrease in PPAR*α* in the TauTKO mouse. Because of the competition between fatty acid metabolism and glucose metabolism, the decline in fatty acid metabolism invariably leads to an increase in glucose metabolism. A key regulatory step in the competition between fatty acid and glucose metabolism is the rate limiting step of glycolysis, phosphofructokinase. Elevations in fatty acid metabolism increase the levels of citrate, an inhibitor of phosphofructokinase. According to Mozaffari et al. the stimulation of glycolysis in the taurine deficient heart is largely mediated by the decrease in citrate content [13]. Also regulating glycolysis in the taurine deficient heart enhanced uptake of glucose from the plasma, an effect directly linked to the activation of phosphofructokinase.

In WT mice, blood glucose levels rise in response to running, an effect likely related to stress-induced elevations in hepatic gluconeogenesis and glycogenolysis. A similar rise in blood glucose levels was observed in the AMPK*α*2 knockdown mouse; however, the rise was attributed to a reduction in glucose uptake by muscle of the AMPK*α*2 knockout mouse [22]. In contrast to the AMPK*α*2 knockout mouse, blood glucose levels remain unchanged in AMPK*β*2 and AMPK*β*1*β*2 knockout mice [24, 32]. Similarly, blood glucose levels remain unchanged in the exercising TauTKO mouse, probably because taurine deficiency and the activation of phosphofructokinase accelerate the uptake of glucose by muscle. Some glucose units in the exercising TauTKO mouse are also derived from muscle glycogenolysis (Table 2), an effect also seen in AMPK*α*2 knockdown mice but not in AMPK *β*2 mice. However, the rate of glycolysis is probably restricted somewhat by the elevation in the NADH/NAD^+^ ratio, which limits flux through glyceraldehyde-3-phosphate dehydrogenase [30]. The increase in the NADH/NAD^+^ ratio also accounts for the rise in the lactate/pyruvate ratio of muscle of the exercising TauTKO mouse.

Pyruvate levels decline in the gastrocnemius of exercising TauTKO mice but not exercising WT mice. On the other hand, gastrocnemius lactate levels in exercising WT and TauTKO mice are similar, revealing a preferential diversion of pyruvate into lactate in exercising TauTKO muscle. Because the gastrocnemius levels of lactate and pyruvate and blood lactate content of resting and exercising WT mice are similar, exercise alone does not affect the utilization of pyruvate by the citric acid cycle. However, both lactate and pyruvate levels decline in the exercising muscle of the TauTKO mouse while blood lactate levels significantly increase, an effect reflecting the significant rise in the NADH/NAD^+^ ratio of TauTKO muscle.

It is interesting that the metabolic pattern of the TauTKO mouse is similar to that of the mitochondrial diseases. A recent study by Jong et al. provides a logical explanation for these observations [17]. Taurine is required for the conjugation of the wobble uridine of tRNA^Leu(UUR)^ [14]. Because the taurine reaction enhances the interaction of the UUG codon with the anticodon of the tRNA, it stimulates the expression of mitochondria encoded proteins whose mRNAs contain a significant number of UUG codons [16]. Indeed, in two of the mitochondrial diseases, MELAS (mitochondrial myopathy, encephalopathy, lactic acidosis, stroke-like episodes) and MERRF (myoclonus epilepsy associated with ragged-red fibers), the conjugation of tRNAs by taurine are impaired by mutations [16, 33]. Characteristic features of the two mitochondrial diseases include decreased respiratory function and ATP synthesis [34, 35]. Muscle weakness is also a characteristic feature of both mitochondrial diseases [36, 37]. However, we failed to observe any changes in the activities of isolated mitochondria between WT and TauTKO muscle (data not shown), while complex 1 activity in skeletal muscle mitochondria of old TauTKO mice was lower than that of WT mice [18]. So far, there are no obvious reports which clarify the physiological role of taurine-conjugated tRNAs in vivo; therefore further studies are necessary to understand the role of taurine conjugation.

Running has no effect on the gastrocnemius ATP content of the WT mouse, largely because regulatory mechanisms are activated that stimulate energy metabolism during stresses, such as vigorous exercise. One of those mechanisms leads to the stimulation of AMPK, which in humans is encoded by seven genes (*α*1,*α*2; *β*1,*β*2; *γ*1,*γ*2,*γ*3), with the catalytic site located on the *α*-subunit and the *β* and *γ* subunits serving regulatory functions [12]. During vigorous exercise, the AMP/ATP ratio of skeletal muscle increases, which promotes the phosphorylation of threonine 172 and activation of the catalytic *α*-subunits of AMPK. Upon being activated, AMPK enhances ATP production of fatigued muscle through the stimulation of both glycolysis and fatty acid *β*-oxidation, the latter through a transcriptional increase in PPAR*α* receptor levels [12, 38]. Key regulatory steps of glycolysis impacted by AMPK include GLUT-4, hexokinase, and phosphofructokinase. AMPK also stimulates fatty acid *β*-oxidation by both increasing fatty acid uptake and indirectly activating carnitine palmitoyltransferase I, a limiting step in the metabolism of long chain fatty acids. Another favorable regulatory mechanism maximizes ATP generation by the respiratory chain through the delivery of more O_2_ to the mitochondria. In the TauTKO mouse both mechanisms are inoperable, as several AMPK subunits (*β*2, *α*1, and *α*2) are downregulated in muscle of TauTKO mice (Figure 3(b)) and the activity of the respiratory chain is diminished [16, 17]. Indeed, the two regulatory mechanisms appear to be related, as respiratory function is also depressed in AMPK*α*2 and AMPK*β*1*β*2 knockout mice [22, 31, 32]. Moreover, the rate of ATP utilization by exercising AMPK*α*2 knockdown mice exceeds the rate of ATP production in the gastrocnemius [22].

A close association exists between AMPK activity and exercise intolerance, as evidenced by increased exercise capacity in animals chronically treated with the AMPK activator, AICAR (5-aminoimidazole-4-carboxamide-1-*β*-D-ribofuranoside) [39], and the decrease in exercise tolerance in AMPK*β*2, AMPK*β*1*β*2, and AMPK*α*2 knockdown or knockout mice [22, 24, 32]. Also supporting the link between AMPK activity and exercise intolerance is the observation that AMPK is downregulated during periods of exercise intolerance [40]. In fact, TauTKO mice fit into the exercise intolerant group. In this regard, the muscle phenotypes of the TauTKO mouse and some of the AMPK knockout models may be similar, as both are characterized by diminished maximal speed and run time during exercise and impaired ability to elevate ATP production during vigorous exercise [12, 24].

Another important function of AMPK is regulation of cell growth, with *α*1 being the subunit most closely tied to changes in muscle size [41, 42]. As the AMP/ATP ratio of skeletal muscle increases and AMPK is stimulated, the myocyte activates ATP-producing pathways while inhibiting ATP-consuming pathways, such as the biosynthesis of proteins involved in muscle hypertrophy [42]. Activation of AMPK with AICAR minimizes muscle hypertrophy during overload stimulation [43]. Conversely, upon overload stimulation AMPK*α*1 knockout mice accumulate more muscle mass than WT mice [42]. Myocytes lacking AMPK are also resistant to Akt-induced hypertrophy [41]. In accordance with the decline in AMPK*α*1, myocyte size is also diminished in the TauTKO mouse [1]. Although further work is necessary to evaluate the effect of AMPK-mediated changes on muscle performance, muscle atrophy likely contributes to the decline in exercise tolerance.

Although most of the genes that are downregulated in TauTKO mice are closely associated with fatty acid metabolism, one of the proteins, glutathione peroxidase 3, regulates oxidative stress. Taurine is not a direct scavenger of reactive oxygen species; however, taurine deficiency promotes the generation of superoxide within the mitochondria by regulating respiratory chain function [17]. The downregulation of glutathione peroxidase 3 may add to the severity of enhanced oxidative stress observed by taurine deficiency. One of the major adverse effects of oxidative stress is the initiation of the mitochondrial permeability transition and cellular apoptosis. Taurine also regulates mitochondrial apoptosis by modulating the formation of the apoptosome [44]. Among other effects, taurine deficiency-mediated apoptosis could contribute to myocyte remodeling in TauTKO muscle.

Several metabolic defects associated with reductions in exercise tolerance have been documented, including the hormone sensitive lipase-null mouse, which is incapable of degrading triglycerides to free fatty acids [45]. In both the hormone sensitive lipase-null mouse and the TauTKO mouse, fatty acid metabolism is severely depressed causing a shift in energy metabolism in favor of anaerobic metabolism. Lipotoxicity, which is a characteristic feature of obesity and diabetes, suppresses contractile function. However, there is no evidence for the accumulation of triglycerides in TauTKO mice [1].

Impaired running endurance has also been detected in mice lacking hepatic glycogen synthase [46, 47]. The liver plays a central role in regulating blood glucose levels through the generation of glucose via gluconeogenesis and glycogenolysis, with glycogenolysis serving as an important source of plasma glucose during acute stress, such as muscle contraction [48].  Table 2 shows that running leads to an extensive decline in hepatic glycogen levels; however, the extent of glycogenolysis is unaffected by taurine deficiency. Although muscle glycogenolysis serves as an important muscular source of glucose during exercise, the data do not support a major role for muscle glycogenolysis in taurine-mediated alterations in energy metabolism or exercise capacity.

In conclusion, our results suggest that taurine depletion in skeletal muscle impairs the regulatory mechanisms that are activated during exercise to facilitate ATP production. As ATP levels fall, the muscle begins to atrophy and the TauTKO mouse eventually becomes exercise intolerant. Contributing to the deterioration in contractile function is the downregulation of several genes, including AMPK, and impaired respiratory function that results in an elevation in the NADH/NAD^+^ ratio and reduced ATP generation. This study shows that taurine plays a critical role not only in excitation-contraction coupling and organelle structure but also in the regulation of skeletal muscle energy metabolism during exercise.

## Figures and Tables

**Table 1:** Primers used for real-time PCR analysis.

Gene	Accession number	Forward primer (5′→3′)	Reverse primer (5′→3′)	Amplicon size (bp)
*Actb *	NM_007393.3	GACAGGATGCAGAAGGAGATTACT	TGATCCACATCTGCTGGAAGGT	142
*Cpt2 *	NM_009949.2	CTGCTCGCTCAGGATAAACAGAA	GGATTGAATGCCATGAATGGA	122
*Echs1 *	NM_053119.2	ATCACCCGGGTCAAGAAACC	TCGCCAGCATAGATGATATCACA	101
*Gpx3 *	NM_001083929.1	GGGCTTCCCTTCCAACCAAT	CCACCTGGTCGAACATACTTGAG	87
*Ppara *	NM_001113418.1	CTGAACATCGAGTGTCGAATATGTG	TGTTCCGGTTCTTCTTCTGAATCT	163
*Prkab2 *	NM_182997.2	GGACGACCCCAGCGTCTT	GGGCGGGCTTCACAGAA	101

**Table 2:** The changes in metabolite and glycogen levels in gastrocnemius muscle and liver before and after exercise in WT and TauTKO mice.

		Muscle ATP (mmol/g wt)	Muscle lactate (nmol/g wt)	Muscle pyruvate (nmol/g wt)	Muscle luc/pyr	Muscle glycogen (mg/g wt)	Hepatic glycogen (mg/g wt)
WT	Rest	2.99 ± 0.053 (5)	62.3 ± 6.65 (6)	1.03 ± 0.13 (6)	77.6 ± 15.8 (6)	1.35 ± 0.098 (7)	40.4 ± 8.09 (7)
	Run	2.96 ± 0.21 (5)	68.6 ± 6.61 (5)	1.25 ± 0.22 (5)	66.7 ± 14.8 (5)	0.99 ± 0.16 (4)^#^	6.78 ± 1.57 (7)^#^

TauTKO	Rest	3.26 ± 0.030 (5)	83.4 ± 5.54 (5)^*^	1.65 ± 0.20 (5)	55.6 ± 5.45 (5)	1.65 ± 0.22 (7)	41.3 ± 6.90 (7)
	Run	2.37 ± 0.31 (8)^#^	70.3 ± 6.21 (8)	0.78 ± 0.15 (8)^#^	113 ± 20.0 (8)	1.02 ± 0.22 (4)^#^	8.83 ± 0.86 (7)^#^

Data are mean ± se. (*n*). ^*^ *P* < 0.05 versus WT, ^#^ *P* < 0.05 versus before running.
